# Electrochemical Formation of Multilayer SnO_2_-Sb _*x*_*O*_*y*_ Coating in Complex Electrolyte

**DOI:** 10.1186/s11671-017-1902-6

**Published:** 2017-02-15

**Authors:** Antonina Maizelis, Boris Bairachniy

**Affiliations:** National Technical University Kharkiv Polytechnic Institute, 21 Frunze Str., Kharkiv, 61002 Ukraine

**Keywords:** Antimony-doped tin dioxide, Pyrophosphate-tartrate electrolyte, Multilayer coating, Electrodeposition, Electrooxidation, 82.45.Yz, 82.45.Fk, 81.16.Rf, 81.15.Pq

## Abstract

The multilayer antimony-doped tin dioxide coating was obtained by cathodic deposition of multilayer metal-hydroxide coating with near 100-nm thickness layers on the alloy underlayer accompanied by the anodic oxidation of this coating. The potential regions of deposition of tin, antimony, tin-antimony alloy, and mixture of this metals and their hydroxides in the pyrophosphate-tartrate electrolyte were revealed by the cyclic voltammetric method. The possibility of oxidation of cathodic deposit consisting of tin and Sn(II) hydroxide compounds to the hydrated tin dioxide in the same electrolyte was demonstrated.

The operations of alloy underlayer deposition and oxidation of multilayer metal-hydroxide coating were proposed to carry out in the diluted pyrophosphate-tartrate electrolyte, similar to the main electrolyte.

The accelerated tests showed higher service life of the titanium electrode with multilayer antimony-doped tin dioxide coating compared to both electrode with single-layer electrodeposited coating and the electrode with the coating obtained using prolonged heat treatment step.

## Background

SnO_2_-Sb _*x*_ *O*_*y*_ coatings are used as a working coatings for sensors [1], supercapacitors [4], electrodes in accumulators [2], fuel cells [3], MEMS microheater devices [5], electrochromatic displays, solar cells, and other optoelectronic devices [6].

Dimensionally stable anodes are used in the processes of electrochemical oxidation of organic substances. Manufacturing such electrodes includes formation coating on the titanium substrate. The coating should have high oxygen evolution overvoltage. There are a few electrodes with high oxygen evolution overvoltage [7, 8]. The lead and tin dioxide coatings are among relatively inexpensive electrode coatings. Besides, the tin dioxide is more effective in reactions of phenol, bisphenol, and aniline oxidation [7].

There are several disadvantages of tin dioxide electrode. It has low conductivity at room temperature, as tin oxide is an n-type semiconducter. It has low stability at anodic polarization. The electrodes with antimony-doped tin dioxide coating (ATO) possess low resistance, high chlorine and oxygen evaluation overvoltage, high exchange current in some test reactions, and phenol removal rate being much higher than the rate on the platinum and lead dioxide electrodes [8]. The oxidation of organic substances on the SnO_2_-Sb _*x*_ *O*_*y*_ is not selective, allowing it to be recommended for multicomponent wastewater purification [9]. The platinum and ruthenium-titanium electrodes accumulate intermediate products during removing of aromatic compounds from wastewaters, while electrochemical destruction on the antimony-doped tin dioxide electrode is full [10].

The active layer of doped tin dioxide can be deposited on a titanium substrate by thermal decomposition of the salt precursor [7, 11], metal sputtering, spin-coating [12], evaporation, ultrasonic spray pyrolysis [13], and the sol-gel method [14, 15]. Coatings with thick layers for dimensionally stable anodes can be obtained by multiple deposition of tin and antimony compounds by dip coating [15–17] or “paint brush” deposition [18] on the titanium substrate with heat treatment of each layer.

The antimony-doped tin dioxide coatings produced with the use of electrochemical stage possess better adhesion to the titanium substrate. Thick ATO coatings are deposited on the electrodeposited underlayer by immersion method with heat treatment of each layer [19–23]. The entire coating can also be fully obtained by electrochemical method followed by thermal oxidation [24–26]. The final stage of nearly all known methods of formation of tin dioxide coating is the prolonged heat treatment at a temperature of 450–600 °C. This stage is associated with significant energy costs. During heat treatment, the titanium dioxide film is formed on the titanium surface. Therefore, despite the fact that the electrochemical step allows obtaining coating with high adhesion to the titanium substrate and the electrodes have a long service life, the electrical resistance still increases during their operation period.

The aim of this investigation was to obtain the antimony-doped tin dioxide coating on titanium substrate by electrochemical method not using durable stage of thermal oxidation of the coating.

## Methods

The investigations were carried out in pyrophosphate-tartrate electrolytes. Composition of the electrolytes is in Table 1. Electrolyte 1 is for tin electrodeposition, electrolyte 2 is for antimony electrodeposition, and the electrolyte 3 is for tin-antimony alloy electrodeposition. The pH of the solutions was 6.5.

**Table 1:** Composition of electrolytes for coating electrodeposition

Components of electrolytes	1	2	3
SnSO_4_, mol L ^−1^	0.3	–	0.3
SbCl_3_, mol L ^−1^	–	0.02	0.02
K_4_P_2_O_7_, mol L ^−1^	0.9	0.9	0.9
KNaC_4_H_4_O_6_, mol L ^−1^	0.1	0.1	0.1
Hide glue, g L ^−1^	1	–	1
Hydrazine, g L ^−1^	10	–	10

The kinetics lows of electrode processes was investigated using potentiostat PI 50-1.1. The results were transferred from analog form to digital by means of the two-channel voltmeter and TeleMax program for PC at a rate of 20 signals per second. Cyclic voltamperograms (CVA) were recorded at the scan rate of 5 mV s ^−1^.

The measurements were carried out in a three-electrode cell. The working electrodes were made of platinum (surface area of 3 cm^2^) and titanium (area of the working surface was 2 cm^2^) plates. The counter electrode was made of platinum (in the case of CVA study) and tin (in the case of metal-hydroxide coating electrodeposition). The saturated Ag/AgCl reference electrode was used in the measurements. Titanium plates were cleaned mechanically, degreased in a 40% potassium hydroxide solution at 80 °C, etched in 15% oxalic acid solution at 98 °C for 2 h, and finally rinsed in distilled water.

The antimony-doped tin dioxide was formed on the titanium substrate in three steps:

1) Electrodeposition of the underlayer of tin-antimony alloy from dilute pyrophosphate-tartrate electrolyte

2) Electrodeposition of tin-antimony metal-hydroxide layer in the main pyrophosphate-tartrate electrolyte (Table 1, electrolyte 3)

3) Electrooxidation of metal-hydroxide layer in the dilute pyrophosphate-tartrate electrolyte

At the first step, the tin-antimony alloy underlayer was deposited from the diluted by five times main pyrophosphate-tartrate electrolyte (Table 1, solution 3). The pH of the dilute solution was 9.0. The current density was 2–3 mA cm ^−2^. Then, the sample was moved to the main operating bath (without rinsing) with the pyrophosphate-tartrate electrolyte. The metal-hydroxide coatings was formed there. After drying the deposit at 100–150 °C, it was oxidized in the diluted electrolyte at the anodic current density of 8–10 mA cm ^−2^.

The content of the metals in the coating was determined my standard titrimetric method: tin content was determined by iodometric titration and antimony content was determined by permanganate titration [27].

The accelerated life test was carried out in 0.5 mol L ^−1^ H_2_SO_4_ solution at the anodic current density of 100 mA cm ^−2^.

## Results and Discussion

### CVA in Pyrophosphate-Tartrate Electrolytes of Sn, Sb, and Sn-Sb Alloy Electrodeposition

The CVA curves in the pyrophosphate-tartrate electrolytes were obtained on platinum by scanning the potential in the cathodic direction first, and then, the deposited layer was oxidized by scanning potential in the anodic direction.

**Fig. 1:**
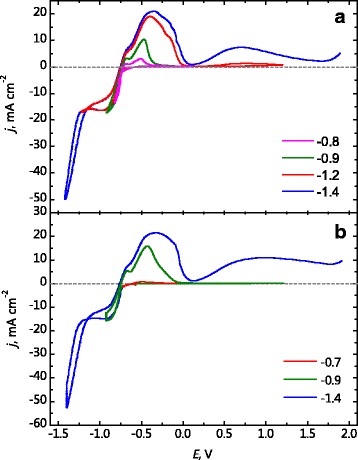
CVA on Pt in the electrolyte for tin deposition with different cathodic borders of the range of potential scan (**a**) and potential delay for 100 s (**b**). The borders of cathodic scanning and potential delay are represented in the figure

Figure 1 a presents the cyclic voltamperograms in the electrolyte for tin deposition(Table 1, electrolyte 1), which were obtained by limiting the cathodic scanning at the more and more negative values of potential. Before −0.7 V tin is not deposited on the platinum electrode, because there are no peaks at the anode branch of CVA. After scanning the potentials up to −0.8 V and up to −0.9 V, two close peaks simultaneously appear on the anode branch of CVA. The quantity of electricity correspondent to the first peak stay almost constant when the cathodic border of the range of potentials scan is getting lower. Presumably, this is due to the formation of tin compounds on the deposit surface. These compounds are the result of the initial dissolution of tin, which was deposited during cathodic period.

The height and area of the second peak increase with increasing of amount of deposited tin. At this peak, tin is dissolved in the form of tin pyrophosphate complexes, in which it is in the oxidation level of + 2. After tin deposition at the limiting current (cathodic border of the range of potential scan is −1.2 V), the shoulder appears on the downward branch of the peak. It is associated apparently with the formation of Sn(II) compounds passivating the surface. At the more positive potentials, the third peak appears on the anodic branch of the CVA curve. According to E-pH diagram at 6.5, only Sn(IV) compounds are produced at the potential range of the third peak [28]. At this peak, the cathodic deposit consisting of tin and its hydroxide oxidizes with formation of Sn(IV) compounds. Both soluble tin pyrophosphate complexes (with tin oxidation level of + 4) and insoluble compounds including tin dioxide can be produced depending on the composition and pH of the electrolyte.

Finally, at potential −1.4 V, tin reduction is accompanied by hydrogen evaluation. The additional quantity of electricity for potential scanning to −1.4 V is almost completely spent for formation of tin hydroxide. This conclusion can be drawn from the fact that the second peak slightly changed, according to the partial current of tin deposition on the cathodic branch, and the third anodic peak substantially increased.

Figure 1 b presents the CVA curves with potential delay at the potentials of cathodic border for 100 s. There were no peaks on the anodic branch of CVA curve after cathodic potential scan to −0.7 V. Though after the potential delay at this potential tin accumulates, that leads to appearance of the second peak on the anodic branch of tin dissolution. The cathodic deposit accumulated for 100 s at −0.9 V does not contain tin hydroxide, because there is no third peak on the anode branch of CVA. After the delay for 100 s at −1.4 V, the third peak exists and it has large area. During the delay at the potential of −0.9 V, the current drops and the coating is light and compact. At the fixed potential of −1.4 V, the current increases indicating that the surface development is due to incorporating of hydroxide compounds.

**Fig. 2:**
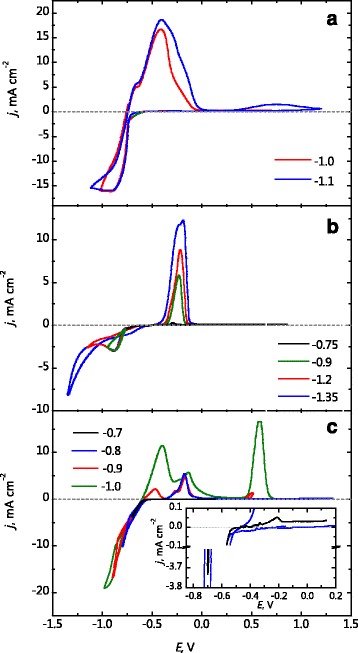
CVA on Pt in the electrolyte for tin (**a**), antimony (**b**), and tin-antimony alloy (**c**) deposition. *Inset* shows the enlarged region of current density. The borders of cathodic scanning are represented in the figure

According to the data at Fig. 2 a, it can be estimated the boundary of the potential scan, after which the process of tin hydroxide compound incorporation begins: after potential scanning in the cathodic direction to −1.0 V, the third anodic peak is absent on the CVA, though after potential scan to −1.1 V, the third peak appears.

Figure 2 b presents the CVA on platinum in electrolyte for the antimony electrodeposition (Table 1, electrolyte 2). Anodic peak of its dissolution appears after potential scanning to −0.75 V. By moving the boundary of the potential scan to more negative potential values, the height of this peak and its area increase. After potential scanning to −1.2 V (where there is the limiting current region and hydrogen starts to evaluate), the shoulder appears on the rising branch of the anodic peak. Due to the hydrogen evaluation during antimony electrodeposition, its hydroxide compounds incorporate into the cathodic coating and current increases at the potential region of shoulder on the anodic peak.

The potential scan to -0.7 V (inset in the Fig. 2c) in the pyrophosphate-tartrate electrolyte for alloy deposition (Table 1, electrolyte 3) shows small peak of antimony oxidation at the anodic branch of the CVA. After potential scanning to −0.8 V (Fig. 2c), anodic peak is also observed only in the region of antimony dissolution potentials. The area under this peak is large due to the larger amount of antimony was deposited at the cathodic period. The coating obtained at −0.9 V already contains tin as the anodic peak on CVA branch presents in region of tin dissolution potentials. Furthermore, the surface Sn(IV) compounds appear in the region of third peak potentials. After potential scanning to −1.0 V, the peaks height of tin oxidation to oxidation level of + 2 and + 4 increase significantly.

### Electrochemical Formation of Antimony-Doped Tin Dioxide on Titanium

**Fig. 3:**
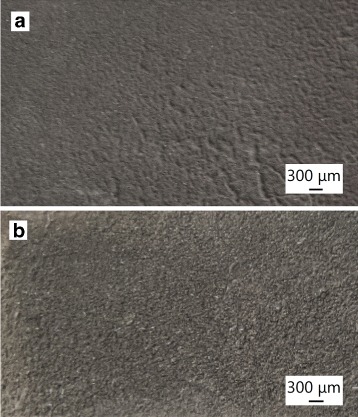
Microphotographs of the surface of ATO of “single layer” type (**a**) and “multilayer” type (**b**)

Two types of antimony-doped tin dioxide coating were deposited. We will refer them “single layer” (Fig. 3 a) and “multilayer” (Fig. 3 b) coatings. Both coatings were deposited on the tin-antimony alloy underlayer to increase the adhesion of the coating to the titanium substrate. For that, the alloy was deposited on the titanium from diluted electrolyte similar to the main bath (Table 1, electrolyte 3). In this diluted electrolyte, the polarization of alloy deposition is higher than the polarization of the main electrolyte. Moreover, the pH of diluted electrolyte was increased to 9.0 raising the part of more stable deprotonated complexes in it. The antimony to tin ratio of the content in the sublayer alloy is 1:16.1.

In the case of the first type of coating, a single-layer coating, the metal-hydroxide layer was deposited from the main bath under galvanostatic regime at the current density exceeding the limiting value for alloy deposition (30 mA cm ^−2^). The antimony to tin ratio of the content in single metal-hydroxide layer is 1:10. In the case of the second type of coating (multilayer), multilayer metal-hydroxide coating with near 100-nm thickness layers was deposited by single-bath technique under galvanostatic two-impulse regime. At the first pulse (25 s) the current density was below the limiting current (10 mA cm ^−2^) leading to electrodeposition of compact alloy layers. At the second pulse (15 s), the current density was higher than the limiting current (40 mA cm ^−2^); therefore, the mixture of metals and hydroxides was deposited. The antimony to tin ratio of the content in the alloy layers of the multilayer coating is 1:8.6, and in the hydroxide layers, the ratio is 1:11.6. For using soluble tin anode in the concentrated electrolyte, the anodic current density must not exceed current value of the second peak of CVA. At the potentials of this peak, tin is oxidized to oxidation level of + 2 in the form of soluble pyrophosphate complexes.

The third stage (electrooxidation of metal-hydroxide coating) of formation of both coating types was carried out in diluted electrolyte again. Previously, the samples with single-layer and multilayer metal-hydroxide coatings were air dried. This results in shifting of their open-circuit potential in diluted electrolyte towards higher values, leaving the region of potentials of active tin dissolution in the form of Sn (II) compounds. In order to allow the target process of tin compound transformation to oxidation level of + 4 in dilute electrolyte, the open-circuit potential of the inserted for anodic treatment samples with metal-hydroxide coating should be above 0.2 V. Electrooxidation was carried out until the open-circuit potential stabilizes in the electrolyte.

### Accelerated Service Life Test of the Antimony-Doped Tin Dioxide Electrodes

**Fig. 4:**
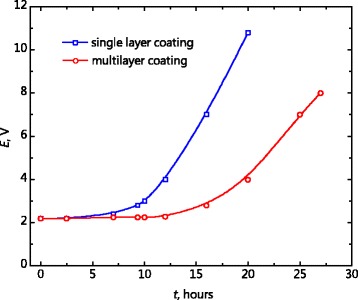
Accelerated service life test curves of electrodes with ATO single- and multilayer coatings in the 0.5 mol L ^−1^ H_4_SO_4_ at the anodic current density of 100 mA cm ^−2^

The time of degradation of the titanium electrode with multilayer antimony-doped tin dioxide coating under condition of accelerated test in the test medium of sulfuric acid is higher compared to both single-layer coating (by 1.5–1.7 times, Fig. 4) and coating obtained using prolonged heat treatment step (by 2–3 times, [19]). This is apparently associated with diminishing of rate of growth of the low-conductivity titanium dioxide film due to the electrodeposition of alloy underlayer. Besides that, the protective properties of the coating become stronger due to presence of alternating layers in the multilayer coating [29] due to the overlapping of the channels (pores) by which the test solution comes to the substrate. Additionally, the multilayer coating is more stable (compared to the single-layer coating) in the slightly alkaline medium during the testing of its catalytic activity in the reaction of phenol oxidation [30].

## Conclusions

The analysis of CVA obtained on platinum in the pyrophosphate-tartrate electrolytes for tin, antimony, and tin-antimony alloy deposition revealed the anodic response to the incorporation of hydroxide compounds in the cathodic deposit in all cases, allowing separation of potential areas of active dissolution of these metals and formation of hydrated oxides on the surface of the coatings. From the electrolyte for the tin-antimony alloy electrodeposition, the antimony deposits at the potential range from −0.7 to −0.8 V. Then, tin incorporates in the deposit, and at the potential of −0.9 V, hydroxides start to include in the coating. Oxidation of Sn (II) compounds to Sn(IV) takes place at the potentials over 0.25 V.

Due to the identity of the qualitative composition of the two electrolytes used, the main and diluted, the steps of coating formation are carried out without intermediate rinsing operations. This saves water and chemicals and reduces the number of operating steps. There is no need in the prolonged heat treatment of the coating that leads to the energy saving in the process of coating formation.

Accelerated life tests showed the higher resistance under anodic polarization of titanium anode with multilayer ATO coating obtained in the pyrophosphate-tartrate electrolyte, compared to both single-layer coating and coatings obtained using the prolonged heat treatment step.
